# Paraneoplastic leukocytoclastic vasculitis as an initial presentation of malignant pleural mesothelioma: a case report

**DOI:** 10.1186/1752-1947-6-261

**Published:** 2012-08-31

**Authors:** Shu Fen Wong, Lisa Newland, Thomas John, Shane C White

**Affiliations:** 1 Austin Health, Department of Medical Oncology, 145 Studley Road, Heidelberg, Victoria, 3084, Australia; 2 Department of Anatomical Pathology, Austin Health, 145 Studley Road, Heidelberg, Victoria, 3084, Australia

**Keywords:** Leukocytoclastic vasculitis, Mesothelioma, Paraneoplastic

## Abstract

**Introduction:**

Vasculitis has been associated with malignancies, more commonly hematological rather than solid malignancies. Due to the rarity of these conditions and the lack of a temporal association, the relationship between vasculitis and malignancy remains unclear. Paraneoplastic vasculitis as a phenomenon of lung cancer has been described in the literature. To the best of our knowledge, this is the first case report of leukocytoclastic vasculitis being an initial presentation of malignant pleural mesothelioma.

**Case presentation:**

We report the case of an 84-year old Greek man who presented to our facility with an erythematous, pruritic and purpuric rash affecting his limbs. This was biopsy-proven to be leukocytoclastic vasculitis and treated conservatively with topical corticosteroids as well as oral prednisolone, with good results. Six months later, he was diagnosed as having malignant pleural mesothelioma. As he remained asymptomatic from his malignancy, no systemic chemotherapy was instituted. He had a recurrence of biopsy-proven leukocytoclastic vasculitis two months after he was diagnosed as having mesothelioma, which again settled with conservative measures.

**Conclusions:**

It is important to remain vigilant with regard to the association between leukocytoclastic vasculitis and malignancies. A diagnosis of vasculitis requires a search for malignancies as well as other possible etiologies. This is particularly of relevance when the vasculitis becomes chronic, recurrent or treatment is no longer effective. Should our patient have experienced refractory vasculitis, we would have instituted systemic chemotherapy to treat the underlying malignancy.

## Introduction

Leukocytoclastic vasculitis (LCV) is a small-vessel inflammatory disease mediated by deposition of immune complexes. It usually occurs in the setting of an underlying process, such as medication exposure, infection, malignancy, connective tissue disease or as a manifestation of a primary systemic vasculitis [1]. Vasculitis has been associated more commonly with hematological rather than solid malignancies [2]. The mechanism by which malignancies cause vasculitis remains unclear. This report illustrates a rare case of paraneoplastic LCV as an initial presentation of malignant pleural mesothelioma (MPM).

## Case presentation

**Figure 1:**
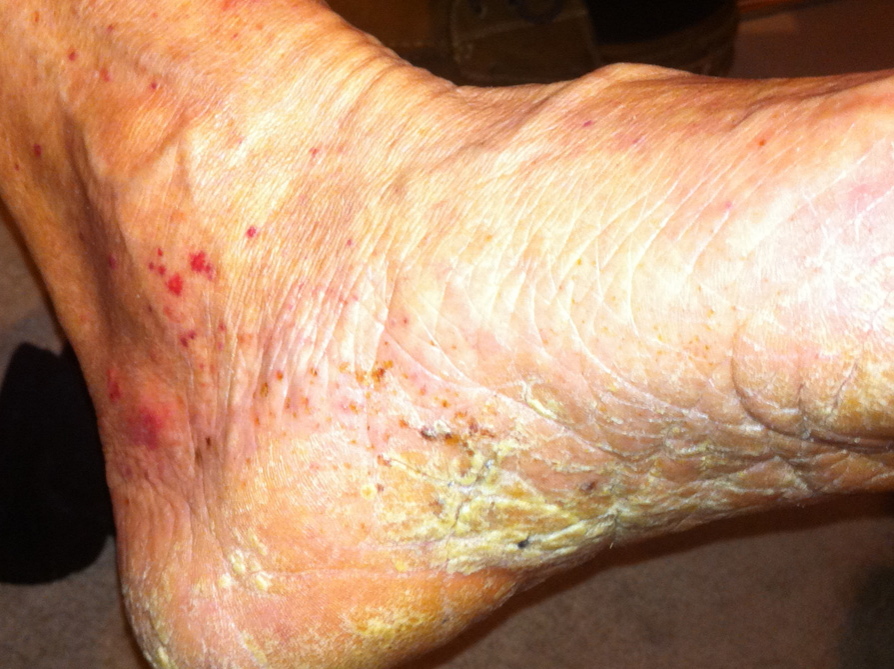
Multiple erythematous, papular and non-blanching rash on lower legs.

**Figure 2:**
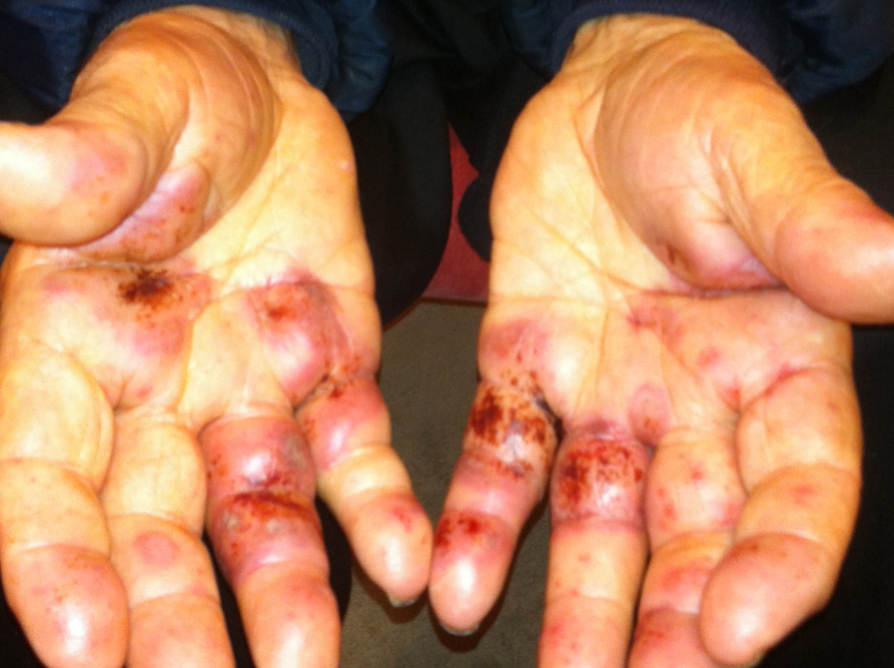
Painful, palpable rash with hemorrhagic bullae of hands.

An 84-year-old previously well Greek man presented to our facility with a one-month history of an erythematous, scaly and pruritic rash that progressed from his left medial malleolus (Figure 1) to involve his lower legs and hands (Figure 2). There were associated superficial erosions and hemorrhagic bullae of the hands, which were painful and interfering with his daily function. His only significant medical history included hypertension and allergic rhinitis for which he was not on medication. There was no history of smoking, asbestos exposure or recent infections.

**Figure 3:**
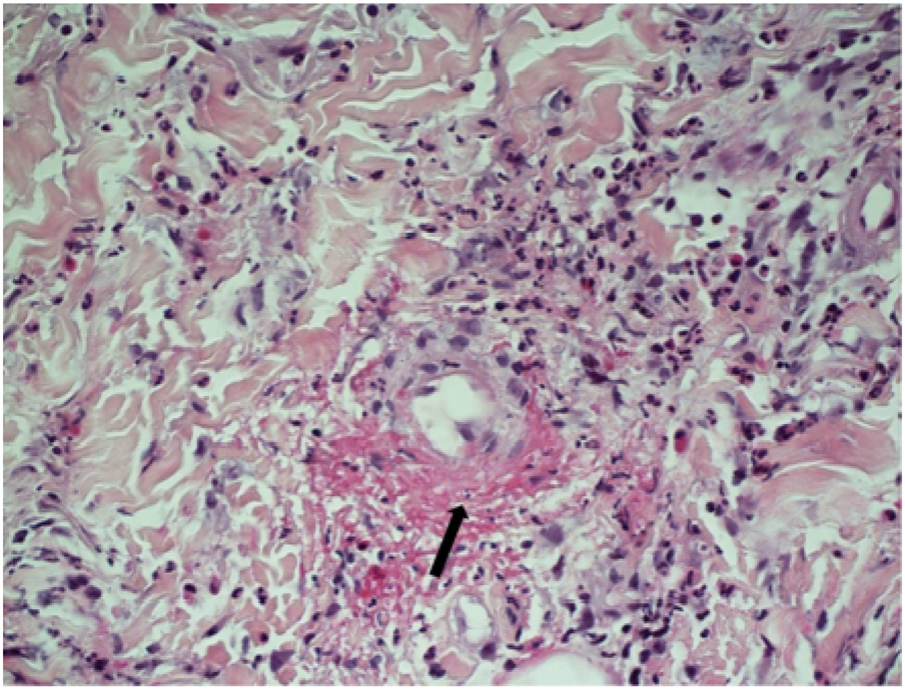
Skin punch biopsy showing features of leukocytoclastic vasculitis with peri-vascular fibrin, neutrophils and leukocytoclasia (nuclear dust), which is indicated with an arrow in the picture (hematoxylin and eosin stain; original magnification×400).

Investigations indicated mild lymphopenia of 0.7 × 10^9^ cells/L (normal range: 1.0×10^9^ cells/L to 4.0 × 10^9^ cells/L), elevated erythrocyte sedimentation rate (ESR) of 85mm/hour (normal range: 5 to 12mm/hour) and a non-specific rise in anti-nuclear antibody of 160U (normal range: <40U). Serum and urine protein electrophoresis, and a chest radiograph were unremarkable. A punch biopsy of a rash on his hand confirmed IgA-positive LCV involving vessels of the superficial and mid dermis, with prominent peri-vascular fibrin, neutrophils and leukocytoclasia (nuclear dust) (Figure 3). Immunofluorescence studies demonstrated IgA and C3 deposition in the walls of superficial vessels. He was treated with oral prednisolone and topical corticosteroids with resolution of the rash.

Six months later, he presented again with a right-sided pleural effusion that contained atypical mesothelial cells on drainage, raising the suspicion of MPM. A subsequent video-assisted thoracoscopic surgery biopsy confirmed biphasic MPM involving the parietal pleura. Laboratory tests demonstrated thrombocytosis of 628 × 10^9^ cells/L (normal range: 150×10^9^ cells/L to 400 × 10^9^ cells/L), elevated C-reactive protein of 293.3mg/L (normal range: <8mg/L) and ESR of 58mm/hour (normal range: 5 to 12mm/hour). A computed tomography scan of the chest revealed right lower lobe collapse and difficulty assessing for a mass lesion.

As he was otherwise asymptomatic, he declined systemic chemotherapy. Two months after being diagnosed as having MPM, he had a recurrence of biopsy-confirmed LCV that again responded to oral prednisolone and topical corticosteroids. The MPM and LCV have not required further treatment, such as chemotherapy, as our patient remains asymptomatic.

## Discussion

LCV is a small-vessel vasculitis featuring leukocytoclasia of infiltrating granulocytes with fibrinoid necrosis of the vascular wall and subsequent extravasation of erythrocytes [3]. The clinical hallmark of LCV includes erythematous macules and urticarial papules, sometimes progressing to palpable purpura, which is usually symmetric but can be diffuse as well [4]. In more than 70% of cases, LCV occurs in the setting of an underlying process inclusive of medication exposure, infection, malignancy, connective tissue disease or as a manifestation of a primary systemic vasculitis [1]. It is classified as idiopathic vasculitis once other etiologies have been excluded.

The occurrence of vasculitis with malignancy has been postulated for many years. It has been estimated that about 5% of patients with vasculitis may have an underlying related malignancy [5]. One retrospective review [6] identified 69 patients with both cancer and malignancy over an 18.5-year period. There were only 12 patients in whom the diagnosis of malignancy and vasculitis occurred within a 12-month period. The most common vasculitis was cutaneous LCV, which occurred in seven cases, with four of them being solid organ malignancies. There was no observed predictable response of vasculitis to treatment with glucocorticoids and systemic chemotherapy. In comparison, a retrospective review conducted by Fain *et al.*[7] identified 60% of their patients (n=60) with vasculitis and malignancy within a 12-month period. LCV was again the most common vasculitis, accounting for 45% of all patient cases. Their review reported one case of mesothelioma associated with vasculitis.

To the best of our knowledge, there have been no previously reported cases of paraneoplastic leukocytoclastic vasculitis occurring with MPM. Our case illustrates that vasculitis may not only be the presenting feature of MPM but may even precede the diagnosis by a number of months. Failure of LCV to respond to usually effective therapies should prompt a search for underlying malignancy. Treatment of paraneoplastic LCV should be directed at the underlying malignancy or by using a combination of corticosteroids and immunosuppresants, especially in the case of hemorrhagic blisters or incipient skin necrosis [3].

## Conclusions

We report a case of paraneoplastic LCV as the initial presentation of MPM. To the best of our knowledge, this is the first case report of LCV presenting with MPM. Corticosteroid treatment dramatically improved the cutaneous LCV in our patient. Should the LCV have been steroid refractory, systemic chemotherapy was considered the next option. However, this has not been required to date and our patient remains well without evidence of progression eight months after diagnosis.

## Consent

Written informed consent was obtained from the patient for publication of this case report and any accompanying images. A copy of the written consent is available for review by the Editor-in-Chief of this journal.

## Competing interests

The authors declare that they have no competing interests.

## Authors’ contribution

SF collected retrospective data from medical records and performed a literature review for the condition. LN analyzed the histological samples and identified illustration samples for the manuscript. SW and TJ reviewed the manuscript and cleaned up the data. All authors reviewed and approved the final manuscript.
